# Intestinal Obstruction by Meckel's Diverticulum in a 92 Years Old Woman

**DOI:** 10.1155/2020/9303059

**Published:** 2020-05-30

**Authors:** Maryam Sarkardeh, Seyed Javad Davari Sani

**Affiliations:** ^1^Department of Surgery, Imam Reza Hospital, Mashhad University of Medical Science, Mashhad, Iran; ^2^Medical Student, Student Research Committee, School of Medicine, Sabzevar University of Medical Sciences, Sabzevar, Iran

## Abstract

**Background:**

Meckel's diverticulum is a vestigial remnant of the omphalomesenteric duct that in most cases (53%) is diagnosed in the first two years of life. *Case report*. A variety of complications are related to Meckel's diverticulum including bleeding, intestinal obstruction, but in adults, the most important complication is intestinal obstruction. We reported Meckel's diverticulum in a 92-year-old woman with symptoms of intestinal obstruction including abdominal pain, vomitus, and abdominal distention who referred to the hospital emergency. Imaging findings showed intestinal obstruction and laparotomy showed that the cause of intestinal obstruction was Meckel's diverticulum. Finally, Meckel's diverticulum was resected and the patient recovered.

**Conclusion:**

Intestinal obstruction because of a large Meckel's diverticulum in an elderly woman is rare and requires a high attention for diagnosis, and it is almost discovered by surgery.

## 1. Introduction

Meckel's diverticulum is a true diverticulum that includes all 3 coats of the small intestine. Meckel's diverticulum is a vestigial remnant of the omphalomesenteric duct [1]. Generally, Meckel's diverticulum ranges from 1-12 cm in length and is found 45-90 cm proximal to the ileocecal valve [2]. Meckel's diverticulum is seen in 2% of the population, and it is the most common congenital malformation of the gastrointestinal tract [3]. Meckel's diverticulum is usually asymptomatic, being found incidentally; however, the main clinical manifestations involve gastrointestinal bleeding, intestinal obstruction, and diverticulitis [4]. In children, gastrointestinal bleeding is the most frequent clinical presentation, while in adults, intestinal obstruction is the most frequent clinical presentation [1]. We presented Meckel's diverticulum in an elderly woman, although most of them (53%) are diagnosed in the first 2 years of life [1].

## 2. Case Report

**Figure 1:**
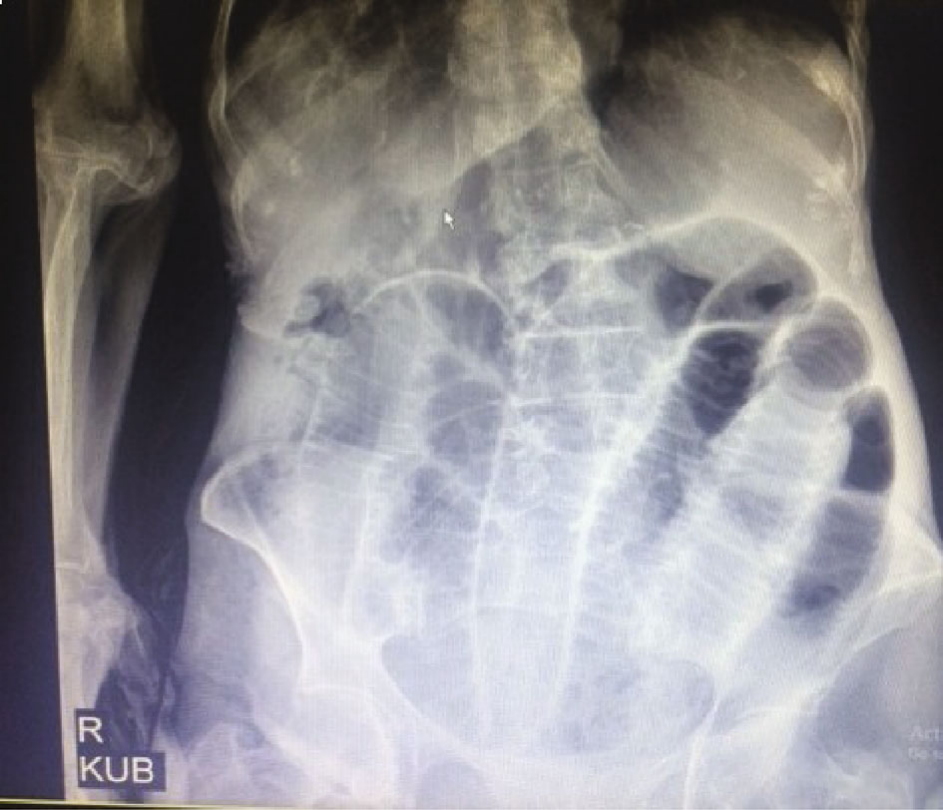
Supine abdominal X-ray.

**Figure 2:**
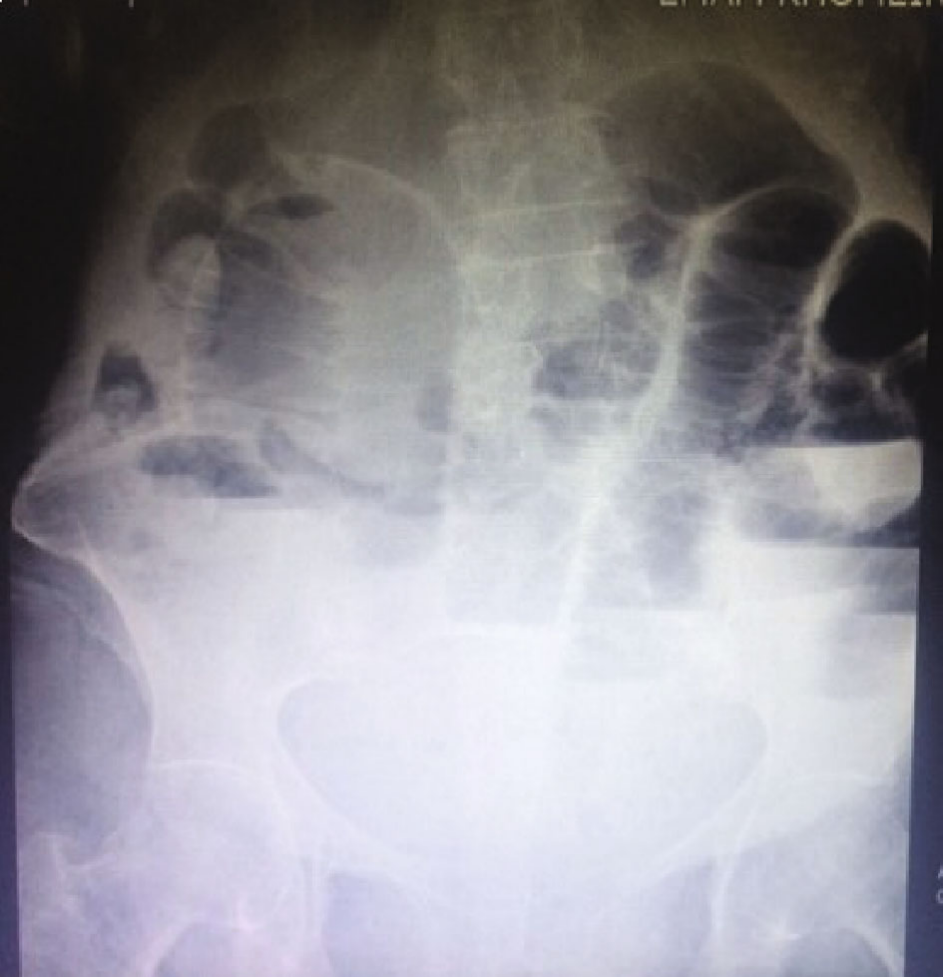
Upright abdominal X-ray.

**Figure 3:**
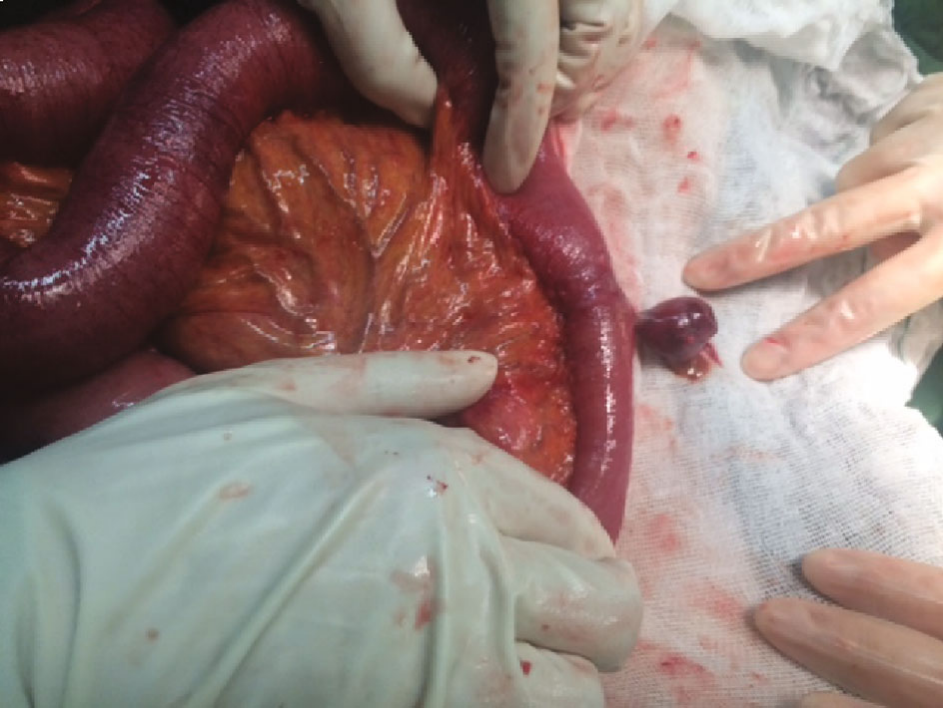
Perforated and narrow based on Meckel's diverticulum.

A 92-year-old woman was referred to the emergent department because of abdominal pain, vomitus, and abdominal distention that had started 3 days ago. The quality of abdominal pain was crampy in the periumbilical area and not radiated to any anatomic region. The last defecation and gas passing was 3 days ago. She had no fever. Her past medical and surgical history was clear. General appearance showed a very old woman and very slim body (weight: 38 kg), with a dry mucosal surface. Vital signs were normal. Other physical examination was norma,l and the abdomen had no tenderness, mass, and hernias on palpation. Also, the rectal exam was empty. Laboratory tests including complete blood count, creatinine, amylase, lipase, electrolytes, and cardiac enzymes were in normal limits. Upright and supine abdominal X-ray showed small bowel with air-fluid levels and dilated bowel loops (Figures 1 and 2). Intravenous fluids were started, nasogastric tube was fixed, and about 600 cc of fecaloid discharge was removed. Because of complete obstruction with fecaloid discharge from nasogastric tube, she was a candidate for laparotomy. The surgery was performed by general anesthesia and a midline incision. Laparotomy revealed dilation of many of intestinal loops and a small internal hernia. At 60 cm from the ileocecal valve, a 5 cm, perforated Meckel's diverticulum was discovered (Figure 3). Other segments of small bowel were normal. Segmental small bowel resection including the diverticulum was performed with a primary end to end anastomosis. The pathology report confirmed Meckel's diverticulum with no heterotopic mucosa.

## 3. Discussion

Meckel's diverticulum is a vestigial remnant of the omphalomesentric duct and is the most common congenital malformation of the gastrointestinal tract [5]. Meckel's diverticulum is so rare over than 50 years. Park et al. in a study of the 1476 cases of Meckel's diverticulum reported that it was discovered at less than 50 years old patients [6]. Similarly, St-Vil et al. in a study of the 164 cases of Meckel's diverticulum reported a mean age of 5.2 years (range, 0 to 18 years) [6]. Christos et al. reported an axial torsion and gangrene of a giant Meckel's diverticulum in a 6-year-old Caucasian boy [7]. There are also other studies of Meckel's diverticulum occured in children, and it is so rare over 50 years. In our case, a Meckel's diverticulum was discovered in a 92 years old woman that it is a rare case. Intestinal obstruction is the most clinical presentation of Meckel's diverticulum in adults, although Meckel's diverticulum is a rare cause of intestinal obstruction in adults [1]. Intestinal obstruction is a major cause of morbidity in hospitals around the world [8]. Etiology of intestinal obstruction is multiple: adhesions (74%), neoplasms (20%), hernias (10%), Crohn's disease (7%), and radiation (1%) [1]. Patients with Chron's disease were younger than patients with other etiologies. The rate of recurrence of intestinal obstruction in patents who had surgery was similar to those who did not undergo surgery [8]. The diagnosis is based on clinical signs, patient history, and imaging finding. Abdominal X-ray with air fluid levels in the small bowel and dilated bowel loops more than 3 cm, and also, trace of gas in the colon is diagnostic [1]. According to technologic developments, abdominopelvic computed tomography (CT) has a 78% to 100% sensibility for complete obstruction, but CT is not useful for detecting Meckel's diverticulum and has little diagnostic value [9, 10]. The management of intestinal obstruction is such that acute and complete obstruction requires immediate surgery, while partial obstruction can be managed conservatively except for an accompanying lesion that requires surgery [10].

## 4. Conclusion

Meckel's diverticulum occurs in children, and it is rare in the adults. The most important complication of Meckel's diverticulum in adults is intestinal obstruction. Imaging findings for the diagnosis of intestinal obstruction due to Meckel's diverticulum have low diagnostic value. Surgery is a diagnostic method of Meckel's diverticulum that presents with intestinal obstruction.

## Data Availability

I would like my article to be available for free.

## References

[1] Capelão G., Santos M., Hilário S., Laureano M., Nobre J., Gonçalves I. (2017). Intestinal Obstruction by Giant Meckel's Diverticulum. *GE-Portuguese Journal of Gastroenterology*.

[2] Holcomb G. W., Murphy J. P., Ostlie D. J. (2014). *Ashcraft’s Pediatric Surgery E-Book*.

[3] Sagar J., Kumar V., Shah D. K. (2006). Meckel's diverticulum: a systematic review. *Journal of the Royal Society of Medicine*.

[4] van Malderen K., Camilleri M. (2018). Large Meckel’s Diverticulum and Dilated Adjacent Small Intestine Presenting With Intestinal Obstruction. *Clinical Gastroenterology and Hepatology*.

[5] Chatterjee A., Harmath C., Vendrami C. L. (2017). Reminiscing on remnants: Imaging of meckel diverticulum and its complications in adults. *American Journal of Roentgenology*.

[6] St-Vil D., Brandt M. L., Panic S., AriéL B., Blanchard H. (1991). Meckel's diverticulum in children: a 20-year review. *Journal of Pediatric Surgery*.

[7] Limas C., Seretis K., Soultanidis C., Anagnostoulis S. (2006). Axial torsion and gangrene of a giant Meckel's diverticulum. *Journal of Gastrointestinal and liver diseases*.

[8] Miller G., Boman J., Shrier I., Gordon P. H. (2000). Etiology of small bowel obstruction. *The American Journal of Surgery*.

[9] Rossi P., Gourtsoyiannis N., Bezzi M. (1996). Meckel's diverticulum: imaging diagnosis. *American Journal of Roentgenology*.

[10] Furukawa A., Yamasaki M., Furuichi K. (2001). Helical CT in the diagnosis of small bowel obstruction. *RadioGraphics*.

